# Radiative Calibration of Heat-Flux Sensors at NIST: Facilities and Techniques

**DOI:** 10.6028/jres.105.033

**Published:** 2000-04-01

**Authors:** A. V. Murthy, B. K. Tsai, R. D. Saunders

**Affiliations:** Aero-Tech, Inc., Hampton, VA 2366; National Institute of Standards and Technology, Gaithersburg, MD 20899-8441

**Keywords:** heat-flux, blackbody, radiometer, sensor, calibration

## Abstract

We present an overview of the National Institute of Standards and Technology high temperature blackbodies, both in operation and in development, suitable for heat-flux sensor calibration. Typical results of calibrations using the transfer technique in the 25 mm Variable-Temperature Blackbody are presented to demonstrate the long-term repeatability of the calibration technique. A comparative study of the absolute and transfer calibrations of a Gardon gage in a spherical blackbody with a cooled enclosure surrounding the gage housing was conducted. Results of this study demonstrated the influence of convection associated with absolute calibration of sensors in a cooled enclosure. Plans for further development of the transfer technique to higher heat-flux levels and the associated technical issues are discussed.

## 1. Introduction

Calibration aspects of heat-flux sensors are not well understood despite their extensive application in aerospace and fire research. Further, the absence of a national standard for heat-flux measurements makes it difficult to provide traceable calibrations at the end-user level. Large variations in the measured responsivity of heat-flux sensors between different manufacturers have been observed in the past. Recognizing these problems and the need to establish traceable calibrations for heat-flux measurements, NIST initiated a competence program in 1995 to develop techniques and establish heat-flux sensor calibration facilities operating on thermal radiation, convection and conduction principles. This paper addresses the radiative calibration aspect of the competence program.

The radiative technique using thermal radiation from a high temperature blackbody is particularly suitable for calibration of heat-flux sensors. This technique has been developed in the Optical Technology Division at NIST to provide calibrations traceable to the High Accuracy Cryogenic Radiometer, the U.S. national standard for optical radiation measurements. The technique, generally referred to as the *transfer calibration* [1], uses blackbody radiation as a transfer source to calibrate sensors with reference to a previously characterized room temperature electrical substitution radiometer, also known as electrically calibrated radiometers. This method has been successfully used in the last three years to calibrate several sensors used as secondary standards for NIST and other agencies up to heat-flux levels of ≈50 kW/m^2^.

While meeting the calibration requirements for other users with the existing capability, the OTD has an on-going research program [2] to provide basic understanding of the various phenomena involved in the heat-flux sensor calibration and to extend the calibration range beyond the present capability. This research program encompasses examination of the relative merits of using different thermal radiation techniques and associated convection problems, and the development of new blackbody facilities. This paper gives an overview of the calibration methodology, and blackbody radiant facilities in use and under development, and discusses related technical issues and plans for further work.

## 2. Calibration Methodology

**Fig. 1:**
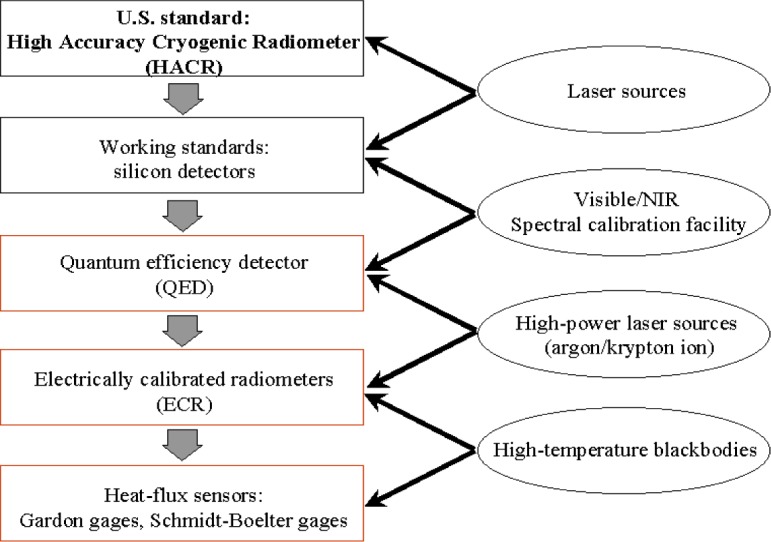
Heat-flux sensor calibration methodology.

The transfer calibration methodology adapted in calibrating heat-flux sensors at NIST is schematically shown in Fig. 1. The methodology establishes a measurement chain resulting in calibrations directly traceable to radiometric standards.

The HACR is the primary standard [3] for optical power measurement in the United States. The HACR is an ECR with a relatively large cavity (time constant ≈240 s) and operates at cryogenic temperatures (≈5 K). The temperature rise in the cavity due to optical power absorption is compared with the electrical power required for the same temperature rise by resistive heating. Operation at cryogenic temperatures considerably reduces the thermal radiation effects due to surroundings and heat dissipation in the wires. These factors lead to highly accurate measurements of the incident optical radiation. The relative expanded uncertainty is 0.02 % (coverage factor *k* = 2 and thus a two standard deviation estimate) at about 1 mW of power, compared to the much larger relative expanded uncertainty of about 1 % for conventional room temperature electrically calibrated radiometers.

The HACR is generally used for calibrating primary working standard trap detectors that are subsequently employed to calibrate working standard photodiodes in the Spectral Comparator Facility. The SCF is comprised of an incoherent source (100 W quartz-halogen lamp) and a prism-grating double-monochromator [4]. The relative expanded uncertainty (*k* = 2) of measurements in the SCF is about 0.22 %.

Quantum efficiency detectors (QED) have an absolute calibration in the wavelength range 406 nm to 920 nm [5]. They can be used as radiometric standards for power measurements. While the QED measurements can be considered absolute, NIST measurements make use of calibration in the SCF to provide traceability to the HACR. However, the maximum power range of the QEDs is limited to about 2 mW and their absolute measurement is valid for a single wavelength of the incident flux. These limitations make the direct application of QEDs difficult for calibrating heat-flux sensors that are used to measure broadband radiation of several orders of magnitude larger in power.

For higher heat-flux levels, cavity type ESRs [6] operating at room temperature serve as suitable transfer standards. These water-cooled radiometers absorb the incident photon flux almost completely because of the blackened walls and multiple reflections within the radiometer cavity. The equivalent electrical power required to produce the same temperature rise in the cavity as the incident flux is determined by heating the cavity by a precision heating element. Considering the effective absorptivity of the cavity and other factors involved in determining the equivalent electrical power, the measurements by these radiometers are likely to be within 0.5 % of the unknown value of the incident flux and can be considered absolute. However, in the NIST measurement chain, instead of using absolute calibration, the ESRs are calibrated against the QED using high-power lasers to provide traceability to the primary standard HACR.

The last step in the chain involves calibration of heat-flux sensors with reference to the calibrated ESR using broadband radiation from a high temperature blackbody. A heated graphite tube variable-temperature blackbody is currently used to provide irradiance levels up to 100 kW/m^2^. This is a variable-temperature blackbody (VTBB) standard source normally used in pyrometric measurements. The ESR and the sensor to be calibrated are exposed to the same level of radiant heat-flux by locating them at a fixed distance from the blackbody aperture. The blackbody is operated at different temperatures to obtain a range of heat-flux levels to calibrate the sensors.

The methodology described above explains the various steps in the calibration of heat-flux sensors, traceable to the U.S. primary standard HACR. Trap detectors calibrated in the HACR can also be used directly to characterize ESR instead of using the QED calibrated in the SCF. This can shorten the measurement chain. It is often the practice to employ the heat-flux sensors calibrated by this approach as reference working standards for routine calibrations of other sensors using less expensive radiant heat sources.

## 3. NIST Radiative Calibration Facilities

Several fixed-point as well as variable-temperature blackbodies and high-power lasers useful in high heat-flux sensor calibration are now operational in the OTD (Table 1). These facilities are described in detail in later sections. For broadband calibration, a large aperture blackbody with variable-temperature capability is preferable. The large aperture blackbody, while providing high heat-flux levels for a given sensor location, also permits placing of sensors inside the blackbody cavity to achieve the highest possible heat-flux levels. Variable-temperature capability provides continuous variation in heat-flux level for a given configuration between the radiating aperture and the sensor. Direct heating by the laser beam is useful in the spectral characterization of reference ESRs that are used as transfer standards in calibrating sensors in blackbody facilities.

### 3.1 25 mm Variable-Temperature Blackbody (VTBB)

**Fig. 2:**
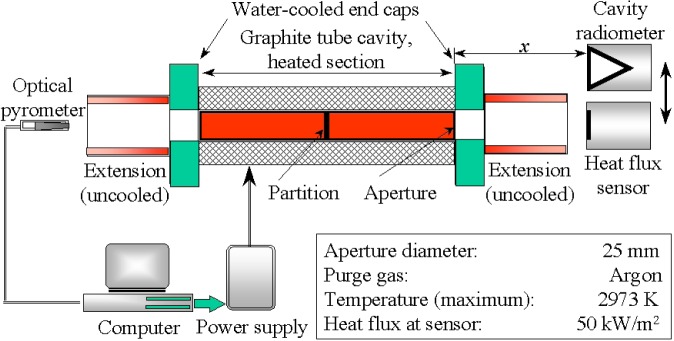
Schematic layout of the NIST 25 mm Variable-Temperature Blackbody.

The 25 mm Variable-Temperature Blackbody^1^ (Thermogage Inc., Frostburg, MD), which is the primary facility used in radiance temperature calibrations, has a large aperture and is particularly suitable for calibrating heat-flux sensors. Since the inception of the competence program, the 25 mm VTBB (Fig. 2) has been extensively used to calibrate sensors and to study various problems related to calibration using blackbody radiation. It is a thermally insulated and electrically heated graphite tube cavity. Direct resistance heating of the tube using large ac currents at low voltages provides for quick heating and cooling. The heated tube cavity diameter is 25 mm, and the heated section is 28.2 cm long with a center 3 mm thick partition.

The tube end caps are water-cooled and are directly connected to the heating electrodes. The design provides a sharp temperature gradient between the end cap and the graphite heater element. This helps in achieving a uniform temperature distribution along the cavity length of the graphite tube. Different lengths of graphite extension tubes can be attached to the end caps. The sensors are placed close to the blackbody exit to achieve the highest possible heat-flux levels.

An optical pyrometer measures the blackbody temperature by sensing radiation from one end of the furnace. A proportional-integral-differential (PID) controller regulates the power supply to maintain the furnace temperature to within ±0.1 K of the set value. The maximum recommended operating temperature for the furnace is 2923 K. The heat-flux sensors to be calibrated and the reference radiometer (ESR) are located at a fixed distance away from the exit of the blackbody. At a distance of 12 mm from the exit, the maximum heat-flux is approximately 50 kW/m^2^ to 60 kW/m^2^. When calibrating at lower heat-flux levels of up to about 10 kW/m^2^, the sensor and the radiometer are located at a distance of about 60 mm from the exit.

### 3.2 Spherical Blackbody

**Fig. 3:**
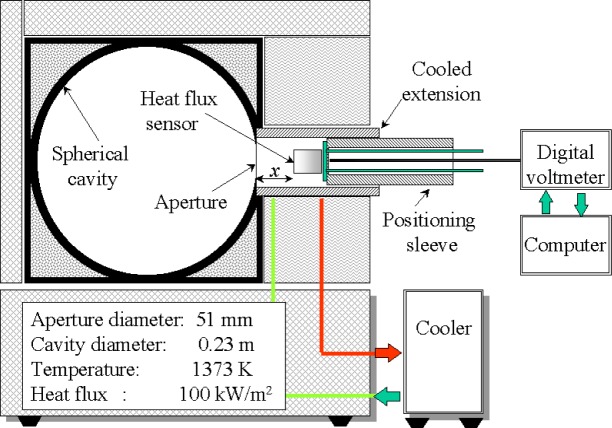
Schematic layout of spherical blackbody with cooled aperture.

The spherical blackbody facility (Fig. 3) was designed and developed recently to study the feasibility of absolute calibrations of sensors in a cooled enclosure. The design was based on the concept first proposed by Olsson [7, 8]. The blackbody cavity is a 0.23 m diameter spherical furnace fitted with a 50.8 mm diameter-radiating aperture^2^.

The spherical furnace wall, made of clay and coated with high temperature black paint on the inner surface, is electrically heated. The exterior of the furnace is air-cooled. The furnace can be operated continuously up to a maximum temperature of 1373 K, and up to 1446 K for shorter duration. A PID controller maintains the cavity temperature, measured by a precision Type-S thermocouple, within ±1 K of the set value. The sensor to be calibrated is located inside a water-cooled enclosure attached to the furnace. The water-cooled enclosure is comprised of a cylindrical tube with a precision aperture at one end, which fits on to the radiating cavity of the furnace. The other end serves as an opening for inserting the sensor housing assembly. The inside of the tube is coated with high temperature black paint. The cooled enclosure minimizes reflected radiation from the inner surface of the tube onto the sensor surface. The test sensor is located inside the enclosure at a fixed distance from the aperture.

Several experimental studies using Schmidt-Boelter and Gardon sensors have been conducted in this facility. These studies demonstrated considerably higher induced flow effects of the hot furnace gas inside the cooled enclosure relative to the VTBB tests. Some of the results of these measurements are presented later.

### 3.3 51 mm Variable-Temperature Blackbody

This facility, commissioned recently, is similar to the 25 mm VTBB (Fig. 1) except for the large diameter cavity and the radiating aperture. However, because of large heat dissipation, a high-pressure closed-loop cooling system is used to provide effective cooling without local boiling. The cooling system consists of a water storage tank, pump/motor assembly and a water-water heat exchanger. The large size of the blackbody cavity facilitates testing of larger size sensors by inserting the sensors directly into the cavity to perform calibrations at the highest possible heat-flux levels. Comparative calibration of a sensor in both the 25 mm and 51 mm facilities is planned to evaluate the relative effects of convection and to provide data useful in determining the effectiveness of computational tools to model the calibration environment.

### 3.4 High-Power Laser Facility

**Fig. 4:**
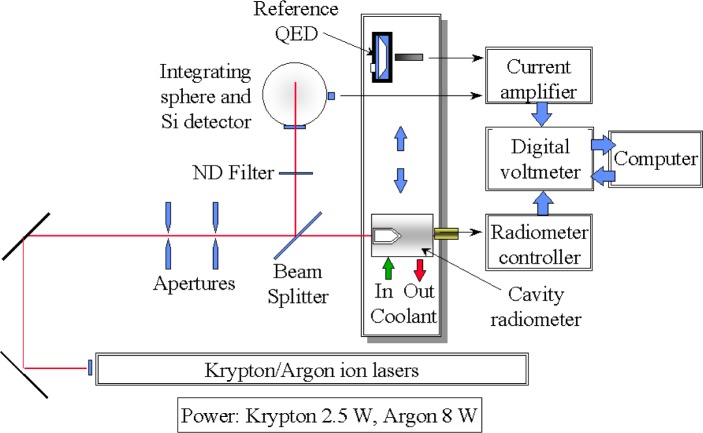
High-power facility for cavity radiometer calibration.

Figure 4 shows schematically the experimental setup in the high-power laser facility used in characterizing transfer standard ESRs with reference to radiometric standards. The facility has two high-power lasers, argon and krypton, lasing at 647.1 nm and 514 nm, respectively.

The laser beam, after being defined by suitable apertures, passes through a beamsplitter. The reference standard QED or the ESR to be calibrated measures the main beam output. The beamsplitter reflects about 8 % of the beam power into an integrating sphere. A silicon detector mounted on the integrating sphere monitors the power of the reflected beam. The silicon detector serves as an intermediate transfer detector to transfer the calibration from the reference optical standard to the heat-flux transfer standard ESR. Currently, NIST has been using a 4.2 W ESR that was calibrated in the laser facility. Further studies are planned with a higher range ESR to extend the range of calibration to higher heat-flux levels.

## 4. Calibration Procedure

First, using the laser facility described in Sec. 3.4, a comparison between a previously characterized QED and the silicon detector is obtained by operating the laser at low power levels. The photocurrent outputs of the QED and the silicon detectors, amplified by low-current amplifiers, are read by a digital voltmeter. Next, the transfer standard ESR is positioned to capture the transmitted beam and the laser operated at higher power levels to cover the full range of the ESR. The corresponding ESR and the silicon detector readings are noted. The laser beam is smaller than the ESR aperture, hence the radiometer measurement represents the full power of the laser beam entering the cavity. For the particular ESR now in use, the aperture area is close to 1 cm^2^, and the radiometer reading also represents the irradiance in W/cm^2^. The ESR calibration is determined by converting the silicon detector output to power using the calibration factor with reference to the QED.

The calibration of the heat-flux sensors with respect to the ESR is done using the VTBB facility. The ESR and the sensor outputs are recorded by operating the VTBB at different temperatures. With blackbody radiation, the ESR aperture is overfilled and the ESR control unit is pre-calibrated to read the heat-flux directly in W/cm^2^. The measurements are done sequentially. The temperature of the VTBB is stable within 0.1 K of the set temperature over the test duration.

## 5. Results and Discussion

Several calibrations and exploratory studies have been made using the facilities. These studies include the NIST transfer technique, feasibility of absolute calibration in the spherical blackbody and the use of other types of radiometers. An overview of the results obtained and the plans for further work are discussed below.

### 5.1 NIST Transfer Technique

One of the requirements in developing traceable calibrations is to establish the long-term repeatability of the technique employed. Also, it is necessary to assess the effect of extraneous experimental factors on the gage calibration. The long-term repeatability of the measurements in the VTBB is checked by calibrating a Schmidt-Boelter gage at frequent intervals. The gage is of miniature type, 5 mm in diameter and 9 mm long. The design heat-flux range is 110 kW/m^2^. In the last 3 years, 13 sets of calibrations have been performed on this gage. These calibrations included different sensor locations with respect to the blackbody aperture, and covered different blackbody temperature ranges.

**Fig. 5:**
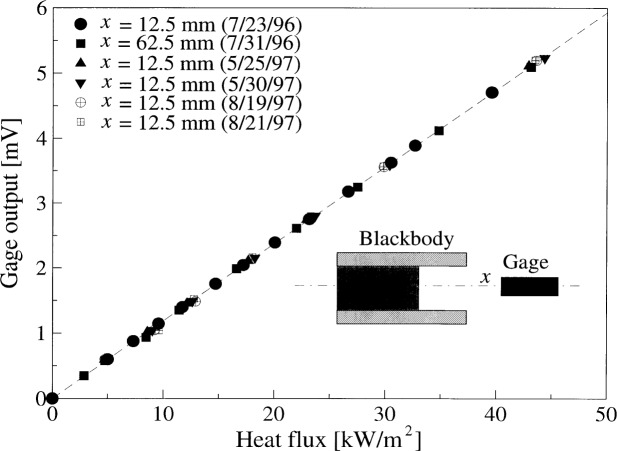
Results of repeat calibrations on a reference Schmidt-Boelter sensor in the NIST 25 mm Variable-Temperature Blackbody. *x*: distance between the blackbody exit and the sensor.

Figure 5 shows the measured response of the gage in millivolts for different levels of incident heat-flux in the range 0 kW/m^2^ to 50 kW/m^2^. All the calibrations show the expected linear response of the gage, with regression factors of unity. Table 2 gives the gage responsivity obtained from linear regression to the measured data. The agreement in responsivity is within approximately ±0.6 % of the mean value from the thirteen calibrations. The good repeatability of the data supports the long-term stability of the transfer standard ESR and the Schmidt-Boelter gage, and validates the OTD measurement technique using the VTBB.

The transfer calibration technique has certain advantages. It is based on direct measures of the heat-flux, rather than blackbody temperature measurements. Hence, any departure of the blackbody radiation from ideal conditions due to extraneous experimental factors will have similar effects on both the reference radiometer and the sensor, and the calibration will not be affected. Further, it is possible to monitor the long-term stability of the reference radiometer with independent calibrations against a radiometric standard.

**Fig. 6:**
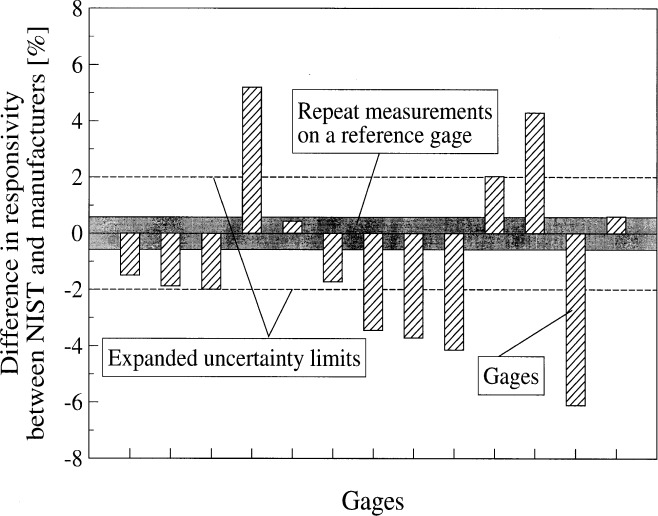
Difference in measured gauge responsivity between NIST and manufacturers’ calibrations.

In the last 3 years, 22 calibrations have been done on different gages using the transfer calibration technique. The calibration range for all the gages was about 50 kW/m^2^. Figure 6 shows the deviation of the manufacturers’ stated responsivity from the NIST calibrations. The assigned relative expanded uncertainty of the NIST calibrations, indicated by dotted lines, is about 2.0 % with a coverage factor *k* = 2. The repeatability of responsivity of the reference gage calibrated over the same time period as the other test gages indicated by the shaded region is within ±0.6 %. In some cases, the manufacturers’ calibrations fall within this range. The long-term repeatability of the reference gage suggests that the present calibration procedure is consistent. The differences of 3 % to 6 % observed between the NIST and manufacturers’ calibrations in some cases are probably due to variations in the calibration methods employed. This broad-based comparison suggests that the present method can provide traceable calibrations with relative expanded uncertainties of 2 %.

### 5.2 Spherical Blackbody Experiments

**Fig. 7:**
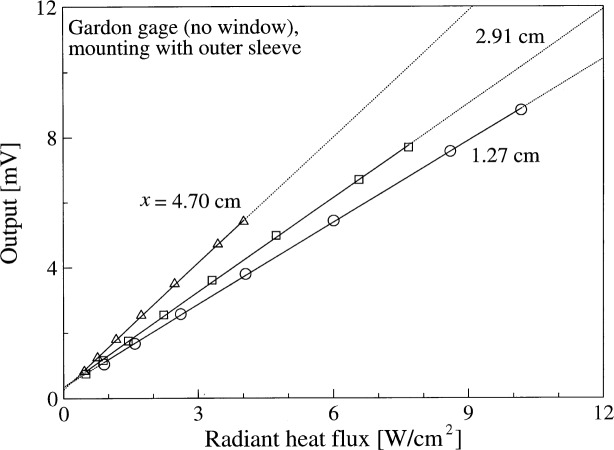
Results of Gardon gauge calibration for radiant heat-flux in the spherical blackbody. *x*: distance between the blackbody aperture and the sensor.

The feasibility of an absolute calibration technique was studied in the spherical blackbody with a cooled enclosure (see Sec. 3.2). Reference [9] gives the details of the experiments and the limitations of using the cooled enclosure technique as an absolute calibrator. For absolute calibration, the heat-flux at the sensor surface is calculated based purely on radiation balance within the enclosure consisting of the blackbody aperture, cooled housing, and the gage assembly. Figure 7 shows the results of the absolute calibration studies on a Gardon gage at three locations of the sensor with reference to the radiating aperture. Contrary to the supposition of reduced convection effects in a cooled enclosure, the studies showed a surprisingly large induced flow effect of the hot gas from the blackbody cavity. The induced flow effect was dominant enough to cause large differences in the calibration depending on the location of the sensor inside the enclosure. The results show the inadequacy of using purely radiant flux calculations for use as an absolute calibrator primary standard. The observed increase in responsivity with distance from the aperture suggests increasing proportion of convective heat-flux relative to radiant flux away from the aperture. It was also noted that the calibration was sensitive to venting of the enclosure to ambient air which caused cooling of the sensor surface [9].

In the present experiments, the aperture and the gage sensitive surface were mounted in a vertical plane (Fig. 3), which results in higher convection effects. It is possible to reduce the convection effects by having the radiating aperture in the horizontal plane and mounting the gage below the radiating aperture of the spherical cavity as in Refs. [7, 8].

The present results demonstrate that for the cooled enclosure technique to be used as an absolute calibrator, it is necessary that the convection effects are kept small. Tilting the test assembly so that the radiating aperture and the test sensor are in a horizontal plane will reduce convection effects to some extent. However, a complete understanding and quantification of the convection effects on the heat-flux at the sensor surface is necessary for the absolute technique to be used successfully as a primary standard.

The cooled enclosure also provides a favorable environment for transfer calibration. The stray radiation from the aperture surroundings incident on the sensor is minimized and convection effects due to purge gas- flow are absent. The convective heat transfer due to induced flow of gas from the furnace cavity will be of similar magnitude for both the reference standard total flux sensor and the test Gardon gage.

For transfer calibration in the spherical blackbody, the reference gage was the Schmidt-Boelter sensor discussed in Sec. 5.1. The measurements were made at five locations. These locations include the three positions = 1.27 cm, = 2.91 cm, and = 4.7 cm from the aperture, discussed earlier. The other two locations were at the aperture plane (0.0 cm) and inside the blackbody cavity at a distance of 0.99 cm from the aperture.

**Fig. 8:**
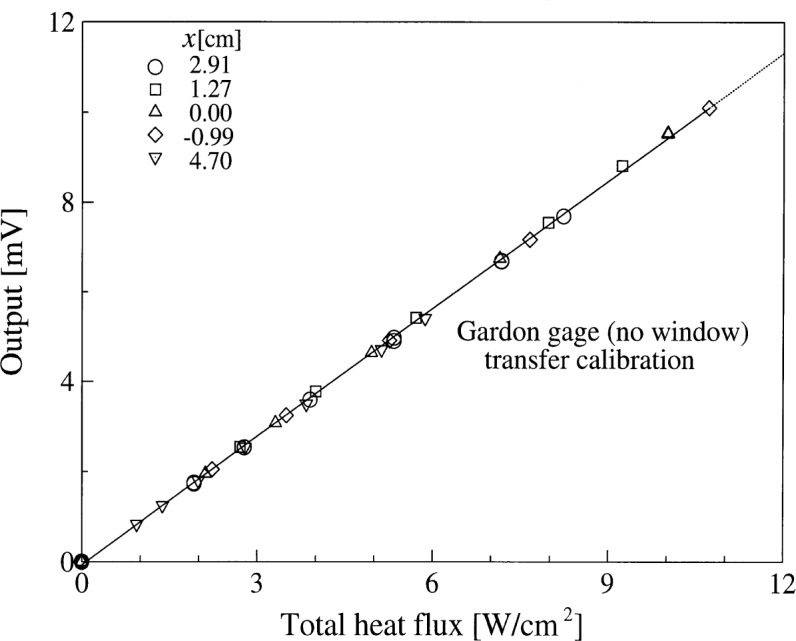
Transfer calibration of Gardon gage with reference to a calibrated Schmidt-Boelter sensor in the spherical blackbody. *x*: distance between the blackbody aperture and the sensor.

Figure 8 shows the results of the transfer calibration of the Gardon gage. The measured responsivity at all five locations agreed within 2 % of the mean value. The mean responsivity obtained from linear regression to the data in Fig. 10 is 0.095 mV/(kW/m^2^), with a relative expanded uncertainty (*k* = 2) of about 3 %. It must be noted that the five locations represent widely varying heat transfer—radiant and convective mix—conditions at the sensor surface.

### 5.3 Calibration at High Heat-Flux Levels

For calibrations at the highest possible heat-flux level corresponding to the blackbody radiation operating at a given temperature, it is necessary to locate the sensor at the blackbody aperture or inside the cavity. The heat-flux at the sensor can be calculated from the Stefan-Boltzmann equation knowing the blackbody temperature and the emissivity. The technique is simple and is widely used. However, the associated convection effects at the cooled surface of the sensor can affect the calibration when not done in vacuum. It is assumed that the convection heat-flux is small in comparison with the radiant heat-flux at the sensor surface. However, no documented evidence appears to be available that demonstrates the relative effects of convection and radiation in such a testing environment.

Particularly when calibrating in high-temperature graphite tube blackbodies, the purge gas flow can affect the heat transfer at the gage surface when the sensor is located close to the blackbody exit. For transfer calibrations in the open mode in the 25 mm VTBB up to about 50 kW/m^2^ heat-flux level at the sensor, the purge gas flow effect appears to be less than 1 % for the final calibration. However, when the sensor is inserted into the cavity, the convection effect can be significant. The magnitude of this effect depends on the internal geometric details of the sensor location inside the cavity and the extent of purge gas-flow. The resulting flow-field around the sensor and the gas temperature in the cavity determine the convection heat-transfer-rate from the sensor surface. Determination of this effect or minimizing it in actual calibration tests remains a challenging problem.

The calibration using the blackbody radiation technique is ideal when carried out in vacuum so that the convection effects at the gage surface are absent. However, when it is not practical, it is necessary to evaluate and, if possible, minimize the convection effect.

### 5.4 Transfer Standard Radiometers

The measurement by the reference standard ESR in the transfer technique represents incident heat-flux in the aperture plane because of total absorption by the cavity. The maximum range for which the transfer calibration can be successfully employed depends on the availability of such a suitable transfer standard with long term repeatability. Present calibrations use an ESR up to about 50 kW/m^2^. Future work is planned with an ESR with heat-flux range up to 200 kW/m^2^. However, the calibration of transfer standard ESRs using laser radiation at higher heat-flux levels may pose problems. The beamsplitter that plays the critical role in transfer ring the calibration from the radiometric standard to the full range of the ESR is prone to develop local hot spots and subsequent damage.

Another type of radiometer receiving considerable attention recently is an ellipsoidal radiometer [10]. This radiometer, first proposed by Gunners [11] in 1967, consists of an ellipsoidal cavity with a highly reflective (gold plated) surface. The aperture opening is located at one focus and the sensing element, which is a typically a Schmidt-Boelter sensor, is located at the other focus. The radiometer is widely in use for furnace and flame radiation measurements since the sensing element is not directly exposed to the hot gases. In contrast to total flux sensors like Gardon and Schmidt-Boelter gages, the ellipsoidal radiometer is not sensitive to convection heat transfer effects. However, unlike ESRs, the ellipsoidal radiometers are not self-calibrating and need to be calibrated with reference to a known standard.

The proposed International Organization for standardization (ISO) International Standard for calibration of heat-flux sensors used in fire testing [10] recommends the use of an ellipsoidal radiometer in the calibration chain. An ellipsoidal radiometer and a total-flux sensor, both calibrated in a primary vacuum facility, serve as primary standards to calibrate secondary-level sensors for both radiation and convection for a specified fire-test configuration. To assess the possible application of ellipsoidal radiometers for NIST calibrations, some preliminary studies were carried out using a commercially available radiometer. The studies consisted of calibrating the ellipsoidal radiometer with reference to the ESR used in the NIST transfer technique. The calibrations were done in the VTBB for different view angles and by direct heating from a high-power argon-ion laser beam.

**Fig. 9:**
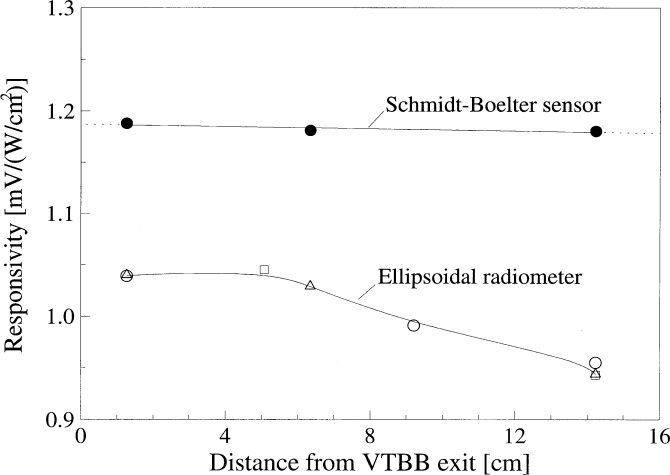
Comparison of ellipsoidal radiometer and Schmidt-Boelter sensor calibrations ian the NIST 25 mm Variable-temperature Blackbody for different locations from the blackbody exit.

Figure 9 shows the calibrations for the ellipsoidal radiometer and also for the reference Schmidt-Boelter sensor discussed in Sec. 5.1. These calibrations were done for different locations from the blackbody exit. The Schmidt-Boelter sensor shows a nearly constant responsivity for various locations from the blackbody aperture. However, the responsivity of the ellipsoidal radiometer decreases gradually away from the blackbody.

This behavior indicates that the responsivity of the ellipsoidal radiometer is a function of the view angle when used in situations other than 180° view. This is probably due to the non-focusing of the incident radiation by the cavity surface to the sensor location inside the radiometer. Because of the imaging characteristics of the reflecting cavity surface, any radiation not focused at the sensor will escape, causing aperture loss. This is illustrated by measuring responsivity by directing a laser beam on different locations of the reflecting cavity surface (Fig. 10a). Three different tests were carried out. In test 1, the laser beam was off-center with respect to the radiometer aperture. In tests 2 and 3, the laser beam was aligned in-line with the radiometer center but the laser beam sizes were different. In all the tests, the laser beam was under-filling the aperture for both the test ellipsoidal radiometer and the reference ESR.

Figure 10b shows the results of laser calibration. It is observed that the responsivity of the test ellipsoidal radiometer increases considerably when the beam is moved towards the center (test 2). In test 3, while the laser beam aligned as in test 2, using an aperture reduced the beam size. The decrease in the beam diameter resulted in still a higher responsivity.

The tests show that the response of the ellipsoidal radiometer is different with laser radiation, hence a direct calibration of the ellipsoidal radiometer with respect to a primary standard like QED using laser radiation is not feasible. When the laser beam diameter is smaller than the sensor located at the focus of the ellipsoid, the distribution of power across the sensor surface is not uniform. The sensor response will be determined more by the peak of the power distribution in the beam. With a larger beam or when the beam is off-center but still under-filling the aperture, some part of the cavity surface close to the sensor will receive radiation. How much of this radiation is reflected to the sensor location is difficult to determine.

The exploratory studies with the ellipsoidal radiometer indicate that the responsivity is a function of the view angle when viewing finite aperture blackbodies. Corrections to the viewing angle have been suggested and radiometers with improved angular response are being considered in the European design [12]. The success of using ellipsoidal radiometers is largely dependent on the outcome of this research.

## 6. Conclusions and Future Plans

An overview of the NIST high temperature blackbodies suitable for heat-flux sensor calibration, both in operation and in development, was presented. The NIST transfer technique using a reference standard electrical substitution radiometer and a variable-temperature blackbody radiator has been successfully implemented to calibrate a number of sensors up to heat-flux levels of ≈50 kW/m^2^. Studies in a spherical blackbody demonstrated that the strong convection effect present in a cooled-enclosure makes it difficult to perform reliable absolute calibration of heat-flux sensors. However, transfer calibration results of a Gardon gage in the spherical blackbody showed good agreement under varying degrees of convection and radiation heat-flux. Plans for extending the calibration capability to higher heat-flux levels and the associated problems have been developed.

## Figures and Tables

**Fig. 10a:**
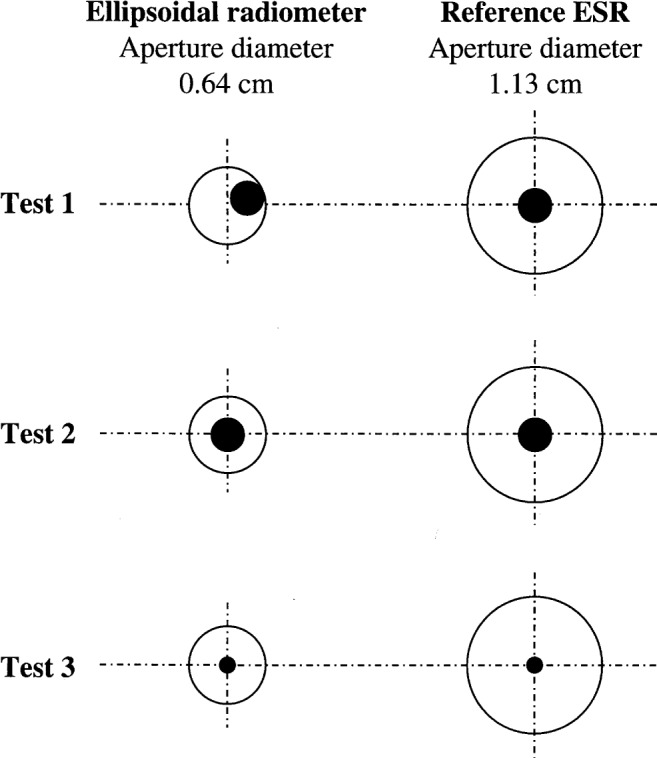
relative position of laser beam inside the ellipsoidal radiometer cavity. Test 1: beam off-center, test 2: beam concentric, test 3: beam concentric and aperture defined. Solid circles represent relative size and location of the beam with respect to the radiometer apertures.

**Fig. 10b:**
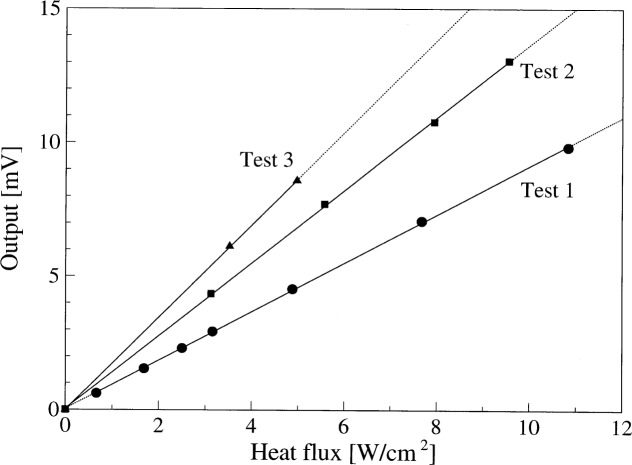
Ellipsoidal radiometer calibration with argon laser for different beam positions inside the radiometer cavity. Measured responsivities are 0.908 mV/(W/cm^2^), 1.360 mV/(W/cm^2^), and 1.727 mV/(W/cm^2^) for test 1, test 2 and test 3, respectively.

**Table 1:** NIST radiative calibration facilities

No.	Facility	Cavity (diameter)	Aperture (diameter)	Heat-flux (Temperature)	Status
1	25 mm Variable-temperature blackbody	cylindrical (25 mm)	circular (25 mm)	3350 kW/m^2^ (2773 K)	Primary facility
2	23 cm Spherical blackbody	spherical (23 cm)	Circular (51 mm)	200 kW/m^2^ (1373 K)	Commissioned in 1997
3	51 mm Variable-temperature blackbody	cylindrical (51 mm)	circular (51 mm)	3350 kW/m^2^ (2773 K)	Available end of 1999
4	High-power argon and krypton lasers.	-NA-	-NA-	up to 8 W	Available

**Table 2:** Measured responsivity of a Schmidt-Boelter heat-flux sensor.

Test No.	*x* [mm]	Responsivity [mV/(kW/m^2^)]	Deviation from mean (%)
1	12.5	0.1189	−0.04
2	62.5	0.1184	−0.46
3	12.5	0.1190	0.10
4	12.5	0.1181	−0.70
5	12.5	0.1191	0.19
6	12.5	0.1197	0.64
7	12.5	0.1200	0.90
8	140	0.1180	−0.79
9	12.5	0.1189	−0.03
10	12.5	0.1199	0.85
11	62.5	0.1187	−0.18
12	12.5	0.1188	−0.13
13	12.5	0.1183	−0.48
Arithmetic mean		0.1189	
Standard deviation		0.0007	0.55
Standard deviation of mean		0.0002	0.16

*x*: distance from VTBB exit to sensor

## Footnotes

1 Certain commercial equipment, instruments, or materials are identified in this paper to foster understanding. Such identification does not imply recommendation or endorsement by the National Institute of Standards and Technology, nor does it imply that the materials or equipment are necessarily the best available for the purpose.

## 8. Appendix A. List of Acronyms

ECR: Electrically calibrated radiometer
ESR: Electrical substitution radiometer
HACR: High accuracy cryogenic radiometer
NIR: Near Infra Red
NIST: National Institute of Standards and Technology
OTD: Optical Technology Division
PID: Proportional – integral – differential
QED: Quantum efficiency detector
SCF: Spectral comparator facility
VTBB: Variable-temperature blackbody
